# Association of HDL2b with chronic kidney disease in type 2 diabetes mellitus populations

**DOI:** 10.3389/fendo.2026.1868445

**Published:** 2026-08-10

**Authors:** Hongjuan Tang, Zilei Ye, Guobao Hong, Xiaohui Li, Yanping Liang, Hequn Zou, David Deng

**Affiliations:** 1 Department of Nephrology, Maoming People’s Hospital, Maoming, China; 2 Depertment of Nephrology, The Third Affiliated Hospital of South Medical University, Guangzhou, China; 3 People’s Hospital of Dianbai District, Maoming, China; 4 Department of Nephrology, The Affiliated Shunde Hospital of Jinan University, Foshan, China; 5 Department of Nephrology, The Chinese University of HongKong, Shenzhen, China; 6 Guangdong Ardent Biomed Co. Ltd, Guangzhou, China

**Keywords:** diabetic kidney disease, HDL2b, high-density lipoprotein cholesterol, inflammation, microalbuminuria, type 2 diabetes mellitus

## Abstract

**Background and purpose:**

Diabetic kidney disease (DKD) is a major microvascular complication of type 2 diabetes mellitus (T2DM). High-density lipoprotein cholesterol (HDL-C) has traditionally been considered renoprotective, yet growing evidence suggests that HDL subclasses may differ in biological function. This study aimed to investigate the association between HDL subclasses, particularly HDL2b, and chronic kidney disease (CKD) in Chinese adults with T2DM.

**Methods:**

In this cross-sectional study, patients with T2DM were enrolled. CKD was defined as urinary albumin-to-creatinine ratio (UACR) ≥3.0 mg/mmol and/or estimated glomerular filtration rate (eGFR) ≤60 mL/min/1.73 m². HDL subclasses were measured using capillary electrophoresis-based microfluidics. Participants were categorized into quartiles of HDL-C, HDL2b, and HDL3. Multivariable logistic regression models were constructed with progressive adjustment for anthropometric indices, hypertension, insulin resistance (HOMA-IR), and inflammatory markers.

**Results:**

Among 481 patients with T2DM, 136 (28.3%) had CKD. Patients with CKD exhibited significantly lower HDL2b levels (P = 0.001), together with greater insulin resistance and systemic inflammation (all P<0.05). HDL2b showed stronger inverse correlations than total HDL-C with adiposity, glycemic indices, insulin resistance, and inflammatory markers (all P<0.05). In multivariable logistic regression, higher HDL2b was independently associated with a lower risk of CKD. Compared with the lowest quartile, the third and fourth quartiles were associated with reduced odds of CKD (Q3: OR = 0.425, 95% CI 0.220–0.821, P = 0.012; Q4: OR = 0.367, 95% CI 0.188–0.717, P = 0.001). The inverse association between HDL2b and CKD was stronger in participants with higher insulin resistance (P for interaction=0.044).

**Conclusion:**

Higher HDL2b, but not total HDL-C or HDL3, is independently associated with lower risk of CKD in T2DM. HDL2b may serve as a sensitive biomarker for early renal risk stratification.

## Background

The prevalence of diabetes mellitus is rising worldwide, representing a major public health challenges in the 21st century (1, 2). In 2019, an estimated 463 million individuals were affected by diabetes globally, and this number is projected to increase to 578 million by 2030 and 700 million by 2045 (3). The development of type 2 diabetes mellitus (T2DM) is typically preceded by prediabetes, an intermediate metabolic state characterized by impaired fasting glucose (IFG), impaired glucose tolerance (IGT) or glycated hemoglobin A1c (HbA1c) levels between 5.7% and 6.4% (4). Prediabetes not only increases the risk of progression to T2DM but also predisposes individuals to diabetes kidney disease (DKD) and cardiovascular disease (5). Among the microvascular complications associated with T2DM, DKD is particularly concerning as it represents a leading cause of end-stage renal disease (ESRD) and is a strong predictor of mortality in this population (2, 6, 7). Urinary albuminuria is defined based on the urinary albumin-to-creatinine ratio (UACR), typically as UACR ≥ 30 mg/g (8). It is a strong and independent risk factor for cardiovascular disease in individuals with T2DM, with risk increasing even at levels below conventional diagnostic thresholds (9). UACR is widely used as an indicator of chronic kidney disease and elevated risk of DKD (10). In addition, urinary microalbumin (UMA), urinary creatinine (UCR), and the UACR are associated with diabetic vasculopathy and diabetic retinopathy (11). Early detection and management of UMA are therefore critical components of comprehensive diabetes care.

High-density lipoprotein cholesterol (HDL-C) has traditionally been considered “good cholesterol” due to its anti-inflammatory, anti-oxidative, and anti-thrombotic properties, which are thought to reduce cardiovascular risk (12). However, observational studies have shown that elevated HDL-C levels are not consistently associated with decreased risk of cardiovascular events (13, 14). Not only in ESRD, but also in early stage of kidney damage, the structure and function of HDL particles as well as HDL subclasses have already become disrupted (15). HDL particles are heterogeneous complexes composed mainly of protein, cholesterol, phospholipids, and triglycerides, with subclass-specific differences in composition. They are synthesized in the liver and intestine and undergo stepwise maturation from precursor HDL to HDL3 and then to larger HDL2 particles through enzymatic cholesterol esterification and lipid and apolipoprotein acquisition (16). HDL particles can be separated by sequential ultracentrifugation into HDL2 and HDL3 on the basis of density, which can be sub-fractionated further into five distinct subclasses (HDL2b, HDL2a, HDL3a, HDL3b, and HDL3c). HDL subclasses are highly heterogeneous in structure, composition and biological activities (17). Among these, the larger HDL2b subclass has been reported to be inversely associated with metabolic syndrome, obesity, insulin resistance, and cardiovascular disease (18, 19). Thus, HDL2b may have potential relevance in assessing chronic kidney disease risk in populations with T2DM.

Previous studies have primarily focused on HDL-C as a global marker, with relatively limited research evaluating specific HDL subclasses, particularly HDL2b, in relation to early kidney dysfunction. Existing evidence indicates that ESRD and coronary artery disease are associated with reduced HDL2b and increased HDL3 levels (20, 21). However, most studies are either conducted in populations without kidney disease or have small sample sizes, limiting generalizability (22). Moreover, there is a paucity of research examining whether HDL2b levels are associated with early renal impairment, as indicated by microalbuminuria, in individuals with T2DM. Understanding this association could provide insight into early pathophysiological changes and help identify novel biomarkers for risk assessment.

Therefore, this study aimed to explore the relationship between HDL subclasses, especially HDL2b, and chronic kidney disease (CKD) in Chinese individuals with T2DM. By simultaneously evaluating HDL subclass profiles, metabolic parameters, inflammatory markers, and renal function indicators, we sought to determine whether HDL2b could serve as a sensitive biomarker for early renal dysfunction and identify individuals at increased risk of developing diabetic kidney disease.

## Methods

### Study design and participants

This was a single-center, observational, cross-sectional clinical study conducted from December 2017 to March 2020 in The Third Affiliated Hospital of South medical University. The criteria for the diagnosis of diabetes are as follows: FPG ≥7.0 mmol/L or 2-h PG ≥11.1 mmol/L during OGTT or HbA1c ≥6.5% or in a patient with classic symptoms of hyperglycemia or hyperglycemic crisis, a random plasma glucose ≥11.1 mmol/L (23). Participants aged ≥18 years with complete clinical, laboratory, and anthropometric data were included. Participants were excluded if they met any one of the following criteria: secondary diabetes, non-diabetic CKD, acute kidney injury, active infection, autoimmune disease, malignancy, recent use of immunosuppressive or lipid-modifying therapies, pregnancy, or inability to comply with study procedures.

The study protocol was approved by the Ethics Committee of The Third Affiliated Hospital of Southern Medical University (approval No. 201708011; approved on August 4, 2017) and was conducted in accordance with the Declaration of Helsinki and applicable ethical regulations. Written informed consent was obtained from all participants before enrolment. This study involved only the collection of clinical data and biological specimens for laboratory testing, and no additional interventions were implemented.

### Data collection and laboratory measurements

First, the participants were asked to complete a questionnaires, including age, gender, smoking, alcohol consumption, physical activity, disease history and family history. Second, with the assistance of medical staff, all subjects completed anthropometric measurements according to the standard program, including body weight, height, waist circumference (WC) and hip circumference (HC). Body mass index (BMI) was calculated as the weight in kilograms divided by the height in metres squared (kg/m^2^). WHR and WHtR were calculated using the following formulas:WHR = WC (cm)/HC (cm); WHtR = WC (cm)/height (cm) (24).

Blood pressure was measured with a calibrated mercury sphygmomanometer after resting for 30 minutes. Venous blood samples were drawn from the participants after overnight fasting. FPG, triglyceride (TG), and total cholesterol (TC) levels were measured by an enzymatic method.

The concentration of HDL-C, very low-density lipoprotein-cholesterol (VLDL-C) and low-density lipoprotein–cholesterol (LDL-C) were measured by a colorimetric method. The level of fasting insulin was measured using an electrochemiluminescence method. The interleukin-6 (IL-6) concentration was measured by a chemiluminescence method. White blood cell (WBC), lymphocyte, neutrophil and monocyte counts were quantified by a Blood Cell Analyzer CAL 8000 (Mindray, China). Homeostatic Model Assessment of Insulin Resistance (HOMA-IR) was calculated as: HOMA-IR = (fasting insulin × fasting glucose)/22.5 (25).

Participants were directed to provide a clean catch, mid-stream urine sample from their first morning void on the day following the visit, and were requested to report the UACR (from the hospital laboratory). Serum creatinine (SCR) were measured by enzymatic method. UMA was measured by immunoturbidimetry. NAG was measured by the MPT substrate method. The estimated glomerular filtration rate (eGFR) (mL/min/1.73 m^2^) was estimated using serum creatinine according to the CKD-EPI equations (26). The CKD in diabetic patients was defined as urinary albumin-to-creatinine ratio (UACR) ≥3.0 mg/mmol and/or estimated glomerular filtration rate (eGFR) ≤60 mL/min/1.73 m^2^ (27).

### HDL subclass measurement

The specific operation method has been described previously (28). HDL subclasses were analyzed using a microfluidic capillary electrophoresis system (MICEP-30; Ardent BioMed, Guangzhou, China) based on molecular size and charge. The assay kit included a microfluidic chip, a polymer-gel separation matrix (linear poly[N,N-dimethyl acrylamide]), specific fluorescent HDL dyes (Dyomics, Jena, Germany), and a microchannel surfactant activator. Serum samples, calibrators, and quality controls were diluted 1:50 in sample buffer (250 mM TAPS, pH 7.5) with a lipophilic fluorescent dye and incubated for 5–15 min, then loaded into chip wells. HDL particles were separated according to their molecular size and charge under an electric field and resolved into five HDL subclasses. Electrophoretic fluorescence signals were analyzed by dedicated software, and HDL2b and HDL3 were quantified as the percentage of each peak area relative to the total HDL peak area. Separation was performed using a preset current and voltage program, and fluorescence was detected at 680 nm. Electrophoretic patterns were analyzed by the system software, and HDL2b and HDL3 percentages were calculated as the proportion of each peak area relative to total HDL. The entire procedure was completed within one hour.

To minimize misclassification among HDL subclasses, quality control procedures were implemented for every analytical run, including one calibrator, one high-level quality control sample, and one low-level quality control sample. Assays were repeated when quality control values fell outside the acceptable range. In addition, all electrophoretic profiles were visually inspected, and samples that failed Gaussian curve fitting were reanalyzed.

### Statistical analysis

Patients were divided into groups according to the presence or absence of CKD. HDL-C, HDL2b, and HDL3 were each categorised into quartiles (Q1-Q4) based on their respective distributions, and comparisons across quartiles were performed using chi-square tests where appropriate.

Statistical analyses were performed using SPSS 23.0 (IBM, Armonk, NY, USA). Significance was set at P < 0.05. Continuous variables were first assessed for normality using the Kolmogorov-Smirnov test. Normally distributed variables are presented as mean ± standard deviation (SD) and were compared between groups using the independent-samples t test. Non-normally distributed variables are expressed as median (interquartile range, IQR) and were compared using the Mann-Whitney U test. Categorical variables are presented as frequencies and percentages and were compared using the chi-square test.

Correlation analyses between HDL subclasses (HDL-C, HDL2b, and HDL3) and metabolic, inflammatory, and renal risk factors were conducted using Pearson or Spearman correlation coefficients, depending on data distribution.

Multivariable logistic regression analyses were performed to evaluate the associations between HDL subclasses and CKD. HDL-C, HDL2b, and HDL3 were entered into the regression models separately and not simultaneously. Odds ratios (ORs) with 95% confidence intervals (CIs) were calculated using the lowest quartile (Q1) as the reference category. Three hierarchical models were constructed: Model 1 was adjusted for age, sex, history of primary hypertension and BMI; Model 2 was further adjusted for HOMA-IR; Model 3 was additionally adjusted for inflammatory markers, including IL-6, hs-CRP, and neutrophil count. Due to non-normal distributions, HOMA-IR, IL-6, hs-CRP, and neutrophil count were natural log-transformed prior to inclusion in the regression models.

Predefined subgroup analyses were performed according to sex, age (<60 vs. ≥60 years), BMI (<24 vs. ≥24 kg/m²), and insulin resistance (HOMA-IR <2.69 vs. ≥2.69). Multivariable logistic regression models were fitted within each subgroup to evaluate the association between HDL2b quartiles and CKD. Interaction terms between HDL2b quartiles and subgroup variables were introduced into the models, and P values for interaction were calculated to assess potential effect modification.

## Results

### Baseline characteristics

As shown in **Table 1**, a total of 481 patients with T2DM were included, of whom 136 had CKD and 345 did not. Patients with CKD were significantly older than those non-CKD (66.46 ± 10.67 vs. 60.09 ± 9.64 years, P<0.001) and had a higher prevalence of primary hypertension (63.2% vs. 40.0%, P<0.001). They also exhibited higher systolic blood pressure (148 ± 18 vs. 140 ± 18 mmHg, P< 0.001). Measures of adiposity, including BMI (26.15 ± 3.90 vs. 25.38 ± 3.41 kg/m², P = 0.015), WHR (0.927 ± 0.06 vs. 0.912 ± 0.06, P = 0.016), and WHtR (0.585 ± 0.07 vs. 0.561 ± 0.06, P<0.001), were significantly higher in the CKD group.

**Table 1:** Characteristics of participants with CKD in T2DM.

Variables	Non-CKD (n=345)	CKD (n=136)	P value
Female,n (%)	136 (39.4%)	51 (37.5%)	0.697
Age (y)	60.09 ± 9.64	66.46 ± 10.67	<0.001
History of primary hypertension, n(%)	138 (40.0%)	86 (63.2%)	<0.001
Blood pressure (mmHg)			
Systolic	140 ± 18	148 ± 18	<0.001
Diastolic	85 ± 9	87 ± 11	0.061
ACEI/ARB drugs, n(%)	6 (6.1)	9 (12.7)	0.174
BMI (kg/m2)	25.38 ± 3.41	26.15 ± 3.90	0.015
WC (cm)	88.82 ± 9.26	91.10 ± 9.03	0.074
HC (cm)	97.35 ± 6.84	98.27 ± 7.07	0.196
WHR	0.912 ± 0.06	0.927 ± 0.06	0.016
WHtR	0.561 ± 0.06	0.585 ± 0.07	<0.001
FPG (mmol/L)	8.02 (7.54-9.10)	8.33 (7.47-10.26)	0.130
OGTT (mmol/L)	8.02 (7.54-9.10)	8.33 (7.47-10.26)	0.030
Fasting Insulin (mmol/L)	11.53 (7.86-15.84)	12.68 (8.90-18.57)	0.035
HOMA-IR	3.78 (2.83-5.67)	5.42 (3.83-8.27)	<0.001
TG (mmol/L)	1.60 (1.20-2.29)	1.69 (1.22-2.46)	0.354
TC (mmol/L)	5.71 ± 1.11	5.48 ± 1.11	0.047
LDL-C (mmol/L)	3.36 ± 1.01	3.22 ± 1.09	0.045
VLDL-C(mmol/L)	0.72 (0.54-1.04)	0.77 (0.54-1.11)	0.409
Apo-A (mg/dL)	1.44 (1.20-1.75)	1.40 (1.20-1.66)	0.139
Apo-B (mg/dL)	0.99 (0.85-1.16)	1.00 (1.83-1.15)	0.438
HDL-C(mmol/L)	1.42 ± 0.35	1.40 ± 0.33	0.068
HDL2b(mmol/L)	0.49 ± 0.01	0.42 ± 0.02	<0.001
HDL3(mmol/L)	0.40 ± 0.01	0.39 ± 0.01	0.646
HDL-C			0.120
Q1	74 (22.9%)	37 (29.4%)	
Q2	88 (25.1%)	40 (26.0%)	
Q3	85 (24.6)	34 (24.9%)	
Q4	98 (27.4%)	25 (19.8%)	
HDL2b			0.001
Q1	63 (19.9%)^a^	46 (36.8%)^b^	
Q2	82 (25.9%)^ab^	36 (28.0%)^ab^	
Q3	80 (25.3%)^ab^	22 (17.6%)^ab^	
Q4	91 (28.8%)^a^	21 (17.6%)^b^	
HDL3			0.738
Q1	87 (25.4%)	26 (19.4%)	
Q2	84 (24.6%)	49 (35.5%)	
Q3	80 (23.1%)	27 (19.6%)	
Q4	88 (25.6%)	35 (25.8%)	
IL-6(pg/ml)	3.44 (2.74-4.63)	4.20 (2.96-6.02)	<0.001
hs-CRP (mg/L)	1.72 (1.09-3.02)	2.13 (1.35-4.16)	0.012
WBC(10^9/^L)	6.70 (5.70-8.20)	7.10 (5.85-8.20)	0.206
Neutrophil (10^9/^L)	3.28 (2.48-4.05)	3.53 (2.90-4.37)	0.003
Lymphocyte(10^9/^L)	2.28 (1.85-2.85)	2.27 (1.77-2.73)	0.251
Monocyte(10^9/^L)	0.49 (0.40-0.64)	0.53 (0.43-0.66)	0.072
Uric Acid(mmol/L)	360 ± 86	383 ± 103	0.009
Creatinine(umol/L)	75.0 (65.75-88.25)	85.0 (71.0-99.50)	<0.001
eGFR(ml/1.72m²)	84.02 ± 12.59	69.14 ± 19.66	<0.001
β2-MG(mmol/L)	0.09 (0.05-0.15)	0.18 (0.08-0.47)	<0.001
NAG(mmol/L)	3.80 (2.20-6.93)	5.40 (2.85-8.55)	<0.001
ACR(mg/mmol)	1.20 (0.85-1.86)	5.26 (3.43-9.96)	<0.001

Data are shown as mean ± standard deviation, median (interquartile range), or frequency (percentage). With each row, different superscripts (a, b) denote P<0.05 corrected by Bonferronic method between groups. Rows sharing the same letter “ab” or with no letters denote no statistically significant difference (adjusted P ≥0.05).

CHD, coronary heart disease; WC, waist circumference; HC, hip circumference; BMI, Body mass index; WHR, Waist-to-hip ratio; WHtR, waist-to height ratio; FPG, fasting plasma glucose; 2-hour OGTT, 2-hour plasma glucose concentration after 75-g oral glucose tolerance; HOMA-IR: Homoeostatic Model Assessment of Insulin Resistance; TG, triglycerides; TC, total cholesterol; ApoA-1, apolipoprotein A-1; Apo-B, apolipoprotein A; VLDL-C: very low-density lipoprotein-cholesterol; LDL-C, low-density lipoprotein-cholesterol; IL-6, interleukin-6; hs-CRP, high- sensitivity C-reactive protein; WBC, White blood cell; eGFR, estimated glomerular filtration rate; β2-MG, Urinary Beta-2-Microglobulin; NAG, N-acetyl-β-D-glucosaminidase; ACR, urine albumin-to-creatinine ratio.

Regarding metabolic and lipid profiles, patients with CKD showed higher OGTT glucose levels (P = 0.030), fasting insulin (P = 0.035), and HOMA-IR (5.42 vs. 3.78, P<0.001), while fasting plasma glucose did not differ significantly (P = 0.130). Total cholesterol and LDL-C were modestly but significantly lower in the CKD group (both P<0.05), whereas TG, VLDL-C, Apo-A, and Apo-B were comparable between groups. HDL2b distribution differed markedly (P = 0.001), bonferroni-adjusted pairwise comparisons indicated that the significant differences were confined to Q1 and Q4. Participants with CKD had a significantly higher proportion in Q1 and a significantly lower proportion in Q4 than those without CKD, while no significant differences were identified for Q2 or Q3. Inflammatory markers, including IL-6 (P<0.001), hs-CRP (P = 0.012), and neutrophil count (P = 0.003), were significantly elevated in the CKD group. As expected, renal injury markers—serum creatinine, uric acid, β2-microglobulin, NAG, and ACR—were significantly higher, while eGFR was significantly lower (69.14 ± 19.66 vs. 84.02 ± 12.59 mL/min/1.73 m², P<0.001).

### Correlation between HDL subclass and CKD risk factor

Correlation analyses demonstrated that HDL-C and HDL2b were broadly and significantly associated with anthropometric and metabolic risk factors related to CKD (**Table 2**). HDL-C showed moderate inverse correlations with BMI (r=-0.245, P<0.001), waist circumference (r=-0.294, P<0.001), WHR (r=-0.236, P<0.001), and WHtR (r=-0.185, P = 0.001). Similarly, HDL-C was negatively correlated with fasting plasma glucose (r=-0.139, P = 0.002), fasting insulin (r=-0.253, P<0.001), and HOMA-IR (r=-0.287, P<0.001). Inflammatory indices including IL-6 (r =-0.200, P<0.001), total white blood cell count (r=-0.251, P<0.001), and monocyte count (r=-0.206, P<0.001) were also inversely associated with HDL-C, whereas no significant correlation was observed with hs-CRP (P = 0.431).

**Table 2:** Correlation between high-density lipoprotein subclass and CKD risk factors.

Variables	HDL-C		HDL2b		HDL3	
	r	P	r	P	r	P
BMI	-0.245	<0.001	-0.364	<0.001	0.157	0.100
WC	-0.294	<0.001	0.380	<0.001	0.156	0.101
HC	-0.220	<0.001	-0.306	0.001	0.108	0.257
WHR	-0.236	<0.001	-0.344	<0.001	0.157	0.099
WHtR	-0.185	0.001	-0.329	<0.001	0.195	0.040
FPG	-0.139	0.002	-0.343	<0.001	0.176	0.064
Fasting Insulin	-0.253	<0.001	-0.205	0.030	0.082	0.390
HOMA-IR	-0.287	<0.001	-0.273	0.004	0.148	0.118
IL-6	-0.200	<0.001	-0.110	0.255	-0.138	0.152
hs-CRP	-0.036	0.431	-0.291	0.002	-0.041	0.667
WBC	-0.251	<0.001	-0.332	<0.001	0.087	0.362
Neutrophil	0.205	<0.001	-0.183	<0.001	0.085	0.377
Monocyte	-0.206	<0.001	-0.016	0.726	0.033	0.731

BMI, Body mass index; WC, waist circumference; HC, hip circumference; WHR, Waist-to-hip ratio; WHtR, waist-to height ratio; FPG, fasting plasma glucose; HOMA-IR: Homoeostatic Model Assessment of Insulin Resistance; IL-6, interleukin-6; hs-CRP, high- sensitivity C-reactive protein; WBC, White blood cell.

HDL2b exhibited stronger and more consistent associations with CKD–related risk factors than total HDL-C. HDL2b was inversely correlated with BMI (r=-0.364, P<0.001), WHR (r=-0.344, P<0.001), WHtR (r=-0.329, P<0.001), fasting plasma glucose (r=-0.343, P<0.001), fasting insulin (r=-0.205, P = 0.030), and HOMA-IR (r=-0.273, P = 0.004). In addition, HDL2b showed significant inverse correlations with hs-CRP (r=-0.291, P = 0.002), WBC count (r=-0.332, P<0.001), and neutrophil count (r=-0.183, P<0.001). In contrast, HDL3 showed no significant correlations with most metabolic or inflammatory parameters, except for a weak positive association with WHtR (r=0.195, P = 0.040), suggesting limited relevance of HDL3 to CKD–related metabolic and inflammatory profiles.

### Association between HDL subclasses and CKD

In **Table 3**, the associations between HDL subclasses and CKD were evaluated across three models with increasing adjustments. For total HDL-C (IQRHDL-C), participants in the highest quartile (Q4) had significantly lower odds of CKD than those in the lowest quartile (Q1) in Model 1 (OR 0.511, 95% CI 0.273-0.955, P = 0.035). However, this association was attenuated after further adjustment for HOMA-IR and inflammatory markers in Models 2 and 3 and was no longer statistically significant. HDL2b showed a consistent inverse association with CKD. Compared with Q1, participants in Q3 had significantly lower odds of CKD across all three models (Model 1: OR 0.358, 95% CI 0.189-0.677, P = 0.002; Model 2: OR 0.371, 95% CI 0.196-0.706, P = 0.002; Model 3: OR 0.425, 95% CI 0.220-0.821, P = 0.012). Similarly, Q4 was independently associated with reduced odds of CKD in all models (Model 1: OR 0.324, 95% CI 0.171-0.611, P = 0.001; Model 2: OR 0.360, 95% CI 0.189-0.685, P = 0.002; Model 3: OR 0.367, 95% CI 0.188-0.717, P = 0.001). In contrast, HDL2b in Q2 was not significantly associated with CKD in any model. No significant associations were observed between HDL3 (IQRHDL3) and CKD. Across all three models, the odds ratios for Q2-Q4 versus Q1 were not statistically significant (all P>0.05).

**Table 3:** Association between HDL subclasses and CKD.

Variables	Model 1		Model 2		Model 3	
	OR (95%CI)	P value	OR (95%CI)	P value	OR (95%CI)	P value
IQRHDL-C						
Q1	Reference		Reference		Reference	
Q2	0.711 (0.402-1.257)	0.241	0.788 (0.440-1.411)	0.432	0.712 (0.379-1.337)	0.291
Q3	0.709 (0.398-1.261)	0.240	0.806 (0.446-1.455)	0.437	0.906 (0.480-1.709)	0.761
Q4	0.511 (0.273-0.955)	0.035	0.619 (0.324-1.181)	0.145	0.575 (0.284-1.167)	0.125
IQRHDL2b						
Q1	Reference		Reference		Reference	
Q2	0.688 (1.177-2.015)	0.552	1.209 (0.702-2.082)	0.493	0.818 (0.438-1.526)	0.220
Q3	0.358 (0.189-0.677)	0.002	0.371 (0.196-0.706)	0.002	0.425 (0.220-0.821)	0.012
Q4	0.324 (0.171-0.611)	0.001	0360 (0.189-0.685)	0.002	0.367 (0.188-0.717)	0.001
IQRHDL3						
Q1	Reference		Reference		Reference	
Q2	1.875 (0.509-6.914)	0.345	1.950 (0.519-7.319)	0.322	2.399 (0.543-10.590)	0.248
Q3	0.172 (0.775-3.506)	0.741	0.791 (0.173-3603)	0.761	0.688 (0.122-3.869)	0.671
Q4	0.994 (0.229-4.309)	0.993	1.070 (0.244-4.689)	0.929	0.667 (0.125-3.562)	0.636

Model 1: adjusted for history of age, sex, primary hypertension and BMI.

Model 2: adjusted for model 1 and HOMA-IR.

Model 3: adjusted for model 2, IL-6, hs-CRP and Neutrophil.

**Figure 1:**
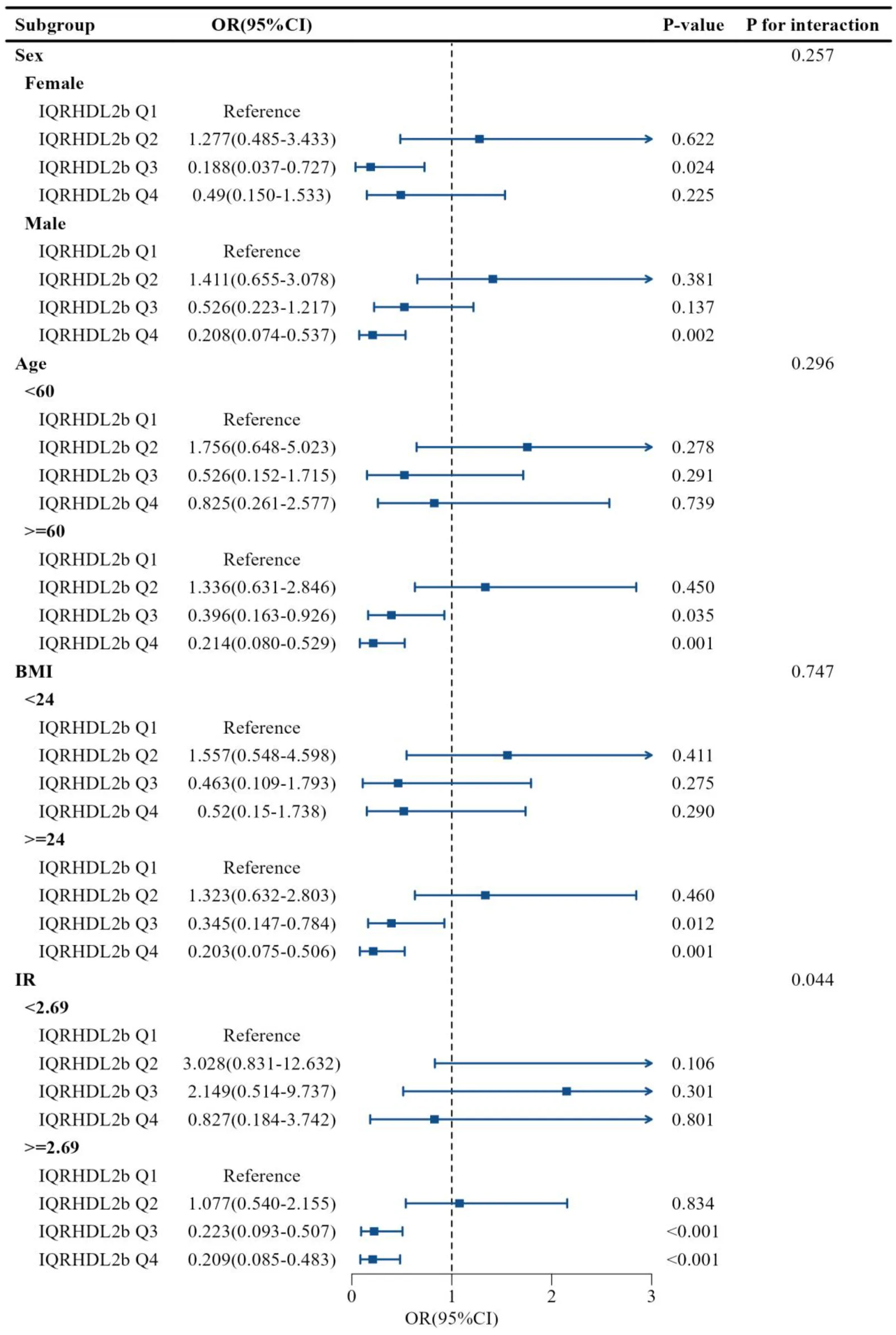
Subgroup analyses of the association between HDL2b quartiles and chronic kidney disease stratified by sex, age, body mass index, and insulin resistance.

Subgroup analyses were conducted according to sex, age, BMI, and insulin resistance (**Figure 1**). The inverse association between HDL2b and CKD was generally consistent across subgroups. Participants in the highest HDL2b quartile (Q4) had significantly lower odds of CKD among males (OR 0.208, 95% CI 0.074-0.537, P = 0.002), individuals aged ≥60 years (OR 0.214, 95% CI 0.080-0.529, P = 0.001), those with BMI ≥24 kg/m² (OR 0.203, 95% CI 0.075-0.506, P = 0.001), and those with HOMA-IR ≥2.69 (OR 0.209, 95% CI 0.085-0.483, P<0.001). No statistically significant associations were observed among participants aged <60 years, those with BMI <24 kg/m², or those with HOMA-IR <2.69. However, no significant interactions were detected for sex (P for interaction=0.257), age (P = 0.296), or BMI (P = 0.747), suggesting that the observed differences between these subgroups were not statistically significant. A significant interaction was observed for insulin resistance (P for interaction=0.044), indicating that the inverse association between HDL2b and CKD was stronger among participants with higher insulin resistance.

## Discussion

In this cross-sectional study of Chinese adults with T2DM, we found that higher levels of HDL2b—but not total HDL-C or HDL3—were independently associated with a lower risk of CKD, defined by microalbuminuria and/or reduced eGFR. Although higher total HDL-C was associated with lower odds of CKD in the minimally adjusted model, this association was attenuated and lost statistical significance after further adjustment for insulin resistance and inflammatory markers. In contrast, the inverse association between HDL2b and CKD remained robust across all multivariable models. HDL3 showed no significant association with CKD. HDL2b levels were inversely correlated with measures of adiposity, insulin resistance, and inflammatory markers, suggesting that this specific HDL subclass may reflect metabolic and inflammatory disturbances that contribute to early renal impairment. These findings highlight HDL2b as a sensitive and clinically relevant biomarker for identifying individuals at heightened risk of DKD at an early stage.

Our results are largely consistent with previous literature indicating that HDL subclasses, particularly HDL2b, are metabolically active and functionally distinct from total HDL-C. Prior studies have demonstrated that HDL2b is inversely associated with obesity, insulin resistance, and the development of cardiovascular disease (29, 30), which mirrors our findings that lower HDL2b levels coincide with higher BMI, WHR, HOMA-IR, and fasting insulin in individuals with T2DM. Notably, although earlier research emphasized total HDL-C as a protective lipid fraction (31), more recent evidence has shown that HDL-C alone does not reliably predict microvascular complications such as DKD (32). In our study, total HDL-C showed a significant inverse association with CKD only in the minimally adjusted model, but this relationship was no longer significant after adjustment for insulin resistance and inflammatory markers. In contrast, HDL2b consistently demonstrated robust inverse associations with CKD across all multivariable models. This divergence underscores the limitations of total HDL-C as a global marker and highlights the clinical relevance of HDL subclass analysis. Additionally, our results suggest that HDL2b may capture subtle metabolic dysregulations that precede overt changes in conventional lipid profiles, potentially providing an earlier signal of renal risk than traditional biomarkers.

Subgroup analyses further demonstrated that the inverse association between HDL2b and CKD was generally consistent across sex, age, and BMI categories, with no significant interactions observed. Although statistically significant associations were observed only among participants aged ≥60 years and those with BMI ≥24 kg/m², the interaction tests suggested that these apparent differences were likely attributable to limited statistical power rather than true effect modification. In contrast, a significant interaction was detected according to insulin resistance status, indicating that the protective association of higher HDL2b was more pronounced among participants with higher insulin resistance (HOMA-IR ≥2.69). This finding is biologically plausible because insulin resistance represents a major driver of diabetic kidney injury through endothelial dysfunction, oxidative stress, chronic low-grade inflammation, and lipid metabolism abnormalities. HDL2b possesses anti-inflammatory, antioxidative, and cholesterol efflux properties, which may become increasingly important under conditions of metabolic stress (33). Consequently, individuals with more severe insulin resistance may derive greater renal protection from higher HDL2b levels, whereas the relatively lower inflammatory and metabolic burden in insulin-sensitive individuals may attenuate the detectable clinical benefit. Nevertheless, given the exploratory nature of these subgroup analyses, these findings should be interpreted cautiously and require confirmation in larger prospective studies.

Inflammation is a key contributor to DKD pathogenesis, and our findings reinforce the interplay between HDL2b and systemic inflammatory processes. Patients with CKD exhibited elevated IL-6, hs-CRP, and neutrophil counts, all negatively correlated with HDL2b but not with HDL3 or total HDL-C. These observations align with prior studies reporting that HDL2b possesses anti-inflammatory and antioxidative properties that modulate endothelial function and immune cell activity (20, 28). Interestingly, while HDL2b levels decreased in conjunction with rising inflammatory markers, HDL3 remained largely unrelated, suggesting functional heterogeneity among HDL subclasses. Mechanistically, reduced HDL2b may impair cholesterol efflux, disrupt endothelial homeostasis, and fail to restrain NF-κB-mediated cytokine release from myeloid cells, thereby accelerating glomerular injury (34, 35). These results extend prior work in metabolic syndrome (36) and CKD populations (16), demonstrating that HDL2b alterations occur early in renal impairment, before ESRD, and may mediate the intersection of metabolic and inflammatory pathways in the progression of kidney disease.

Compared with studies in ESRD or advanced CKD populations (9, 37), our cohort predominantly represents patients with earlier-stage CKD, emphasizing the potential of HDL2b as a biomarker for early renal dysfunction rather than advanced disease. Previous research has often focused on severely dyslipidemic or uremic patients, where HDL dysfunction is widespread; in contrast, our study captures the preclinical phase of kidney injury, where interventions may be most effective (38, 39). The lack of association between HDL3 and CKD highlights the importance of HDL particle heterogeneity: HDL3 particles are smaller, denser, and compositionally distinct, with less influence on glucose and lipid metabolism or endothelial protection (40). Our results suggest that subclass-specific measurement, particularly of HDL2b, provides a more nuanced assessment of metabolic and inflammatory stress than total HDL-C or HDL3, offering potential utility in risk stratification and early intervention among diabetic populations.

Potential mechanisms linking HDL2b to renal protection involve modulation of insulin resistance, inflammation, and endothelial function (41). Insulin resistance contributes to hyperglycemia, endothelial dysfunction, glomerular hyperfiltration, and albuminuria (42), while HDL2b may counteract these processes by promoting cholesterol efflux, enhancing β-cell insulin secretion, and suppressing pro-inflammatory signaling (43). Furthermore, HDL2b may interact with myeloid cells and mineralocorticoid receptor (MR) signaling pathways, reducing oxidative stress, inflammatory mediator release, and fibrotic signaling in the kidney (44, 45). These mechanistic pathways are supported by preclinical evidence showing that MR activation in myeloid cells exacerbates renal inflammation (46, 47), while HDL2b exhibits anti-inflammatory and anti-oxidative effects capable of mitigating these processes. Clinically, our findings suggest that HDL2b assessment could improve early DKD risk stratification, guide therapeutic decisions, and potentially serve as a surrogate marker for monitoring intervention efficacy, thereby enhancing precision medicine strategies for T2DM.

## Limitation

Several limitations should be noted. First, this was a single-center cross-sectional study, which limits causal inference and generalizability to broader populations. Second, although we measured HDL2b and HDL3 using a validated microfluidics system, other HDL subclasses (e.g., HDL2a, HDL3a-c) were not assessed due to stability and reproducibility concerns under routine laboratory conditions. Evaluating the full spectrum of HDL subclasses may provide additional insights into their differential roles in CKD. Third, although we adjusted for multiple metabolic and inflammatory factors, residual confounding from unmeasured variables such as diet, physical activity, or medication adherence may persist.

## Conclusion

In summary, HDL2b is independently and inversely associated with CKD in adults with T2DM, while total HDL-C and HDL3 show no significant relationship. HDL2b levels correlate with insulin resistance, adiposity, and inflammation, supporting their role as a sensitive biomarker for early renal impairment. Measurement of HDL2b may enhance risk stratification and guide early interventions to prevent the progression of diabetic kidney disease.

## Data Availability

The original contributions presented in the study are included in the article/supplementary material. Further inquiries can be directed to the corresponding authors.
